# Prediction of early graft function after living donor kidney transplantation by quantifying the “nephron mass” using CT-volumetric software

**DOI:** 10.3389/fmed.2022.1007175

**Published:** 2022-10-28

**Authors:** Kazuhiro Takahashi, Kinji Furuya, Masahiko Gosho, Joichi Usui, Tomokazu Kimura, Akio Hoshi, Shinji Hashimoto, Hiroyuki Nishiyama, Tatsuya Oda, Kenji Yuzawa, Kunihiro Yamagata

**Affiliations:** ^1^Department of Gastrointestinal and Hepato-Biliary-Pancreatic Surgery, University of Tsukuba, Tsukuba, Japan; ^2^Department of Biostatistics, University of Tsukuba, Tsukuba, Japan; ^3^Department of Nephrology, University of Tsukuba, Tsukuba, Japan; ^4^Department of Urology, University of Tsukuba, Tsukuba, Japan; ^5^Department of Transplant Surgery, Mito Medical Center, Mito, Japan

**Keywords:** cortex weight recipient weight ratio, estimated glomerular filtration rate (eGFR), living-donor kidney transplantation (LDKT), multidetector raw CT (MDCT), weight ratio (WR)

## Abstract

Early renal function after living-donor kidney transplantation (LDKT) depends on the “nephron mass” in the renal graft. In this study, as a possible donor-recipient size mismatch parameter that directly reflects the “nephron mass,” the cortex to recipient weight ratio (CRWR) was calculated by CT-volumetric software, and its ability to predict early graft function was examined. One hundred patients who underwent LDKT were enrolled. Patients were classified into a developmental cohort (*n* = 79) and a validation cohort (*n* = 21). Using the developmental cohort, the correlation coefficients between size mismatch parameters, including CRWR, and the posttransplantation estimated glomerular filtration rate (eGFR) were calculated. Multiple regression analysis was conducted to define a formula to predict eGFR 1-month posttransplantation. Using the validation cohort, the validity of the formula was examined. The correlation coefficient was the highest for CRWR (1-month *r* = 0.66, *p* < 0.001). By multiple regression analysis, eGFR at 1-month was predicted using the linear model: 0.23 × donor preoperative eGFR + 17.03 × CRWR + 8.96 × preemptive transplantation + 5.10 (adjusted coefficient of determination = 0.54). In most patients in the validation cohort, the observed eGFR was within a 10 ml/min/1.73 m^2^ margin of the predicted eGFR. CRWR was the strongest parameter to predict early graft function. Predicting renal function using this formula could be useful in clinical application to select proper donors and to avoid unnecessary postoperative medical interventions.

## Introduction

In most patients undergoing living-donor kidney transplantation (LDKT), immediate graft function (IGF), in which grafts show immediate urinary formation, is observed (1, 2). The serum creatinine level declines rapidly, and the level reaches nadir within a few weeks posttransplantation. The loss of nephrons owing to immunological and non-immunological factors reduces graft function on a yearly basis, and eventually, the function of the transplanted kidney is abolished (3–5). Early posttransplantation renal function affects subsequent renal function and long-term graft survival (6, 7). Therefore, maintaining the “nephron mass” of the renal grafts is essential for long-term graft survival. Owing to the progress in immunosuppressive therapy and the establishment of a highly accurate diagnostic and elaborate treatment strategy against immunological complications, the 5-year graft survival rate now reaches 90% in most countries (1, 8, 9).

Initial renal function after LDKT is affected by donor age, sex, donor preoperative estimated glomerular filtration rate (eGFR), donor-recipient size difference, etc., all of which reflect the “nephron mass” of the renal graft (10, 11). The donor-recipient size mismatch is manifested by the clearance capacity of the “nephron mass” in the donor graft, which is less than the recipient’s metabolite production. Because the “nephron mass” cannot be directly measured, alternative parameters that can be used as indicators of donor-recipient size mismatch include the donor to recipient weight ratio (WR), body mass index (BMI) ratio (BMIR), body surface area (BSA) index ratio (BSAR), actual graft weight (Graft-act) to recipient weight ratio (GRWR-act), and Graft-act to recipient BSA ratio, and the relationship between these parameters and posttransplantation renal function has been discussed in the previous literature (12–16). On the other hand, with the recent developments in medical technology, the “nephron mass” can be measured directly by quantifying the renal cortex using 3-dimensional (3D) CT-volumetry based on contrast-enhanced images obtained by multidetector raw CT (MDCT) (17).

The purpose of this study was to quantify the “nephron mass” of the renal graft using CT-volumetric software and examine whether our novel size mismatch parameter that directly reflects the “nephron mass,” the cortex to recipient weight ratio (CRWR), could determine early renal function after LDKT compared with other representative size mismatch parameters.

## Materials and methods

### Study population

Between October 2013 and February 2022, 112 patients underwent ABO identical/compatible adult-to-adult LDKT at Tsukuba University Hospital (*n* = 85) and Mito Medical Center (*n* = 27). Patient records were identified by an administrative database. In both cohorts, all LDKTs were conducted with Asian pairs of donors and recipients, and none of the recipients demonstrated delayed graft function. Eight patients were excluded since the arterial phase of the contrast-enhanced CT was insufficient for reconstruction. Four patients were excluded since the patients demonstrated acute rejection within 12 months posttransplantation. Thus, a final population consisting of 100 pairs of LDKT was enrolled in our study. Patients who underwent transplantation at Tsukuba University Hospital (*n* = 79) were allocated as a developmental cohort to build a prediction model, and patients who underwent transplantation at Mito Medical Center (*n* = 21) were allocated as a validation cohort. All data for this study were collected in accordance with the Tsukuba University Hospital and Mito Medical Center Internal Review Boards.

### Immunosuppression

In both centers, basiliximab (20 mg/body) was introduced on the day of surgery and 4 days after surgery. Maintenance immunosuppression was conducted with triple therapy comprising long-acting tacrolimus, mycophenolate mofetil, and steroids. The trough value of tacrolimus was maintained at 7–10 ng/mL 3 months after surgery and 5–8 ng/mL 4–12 months after transplantation.

### CT-volumetric quantification

The Digital Imaging and Communications in Medicine data were obtained from MDCT with 1–2 mm slices (Brilliance 64 multidetector row CT scanner, Philips, Netherlands) and transferred to high-end simulation software (Synapse Vincent^§^ ver. 6.1; Fujifilm Corporation, Tokyo, Japan), which semiautomatically calculates the volume of the kidney graft and its cortex (Supplementary Figure 1). The collecting system, vessels, cysts, and sinus were excluded from all parenchymal volume measurements. Graft-act was measured immediately after the back-table procedure. The simulated graft weight (Graft-sim) to recipient weight ratio (GRWR-sim), CRWR, and GRWR-act were defined as Graft-sim divided by the recipient body weight (before LDKT), calculated cortex volume divided by the recipient body weight, and Graft-act divided by the recipient body weight, respectively. For the comparison between predicted kidney volume and actual kidney graft weight, we defined 1 g of kidney tissue as having a volume of 1 ml.

### Assessment of renal function and urinary protein

Graft function was evaluated based on eGFR (ml/kg/1.73 m^2^), calculated using the conversion formula for Japanese individuals. Proteinuria was measured in 24-h urine samples. The donor eligibility criterion at both centers was renal function with an inulin clearance of at least 70 ml/min. In this study, since postoperative renal function was assessed by eGFR, eGFR was also used for preoperative renal function.

### Glomerular morphometry of renal biopsy specimens

All protocol renal biopsy samples were obtained at 12 months posttransplantation using a percutaneous needle device. A 3 mm section of paraffin-embedded renal cortex specimen from the recipient was stained with hematoxylin-eosin. Glomerular area was measured by tracing the contour of the outer margins along the glomerular tufts using imaging software (BZ-X Analyzer, Keyence, Osaka, Japan) (Supplementary Figure 2). The volume of three glomeruli or more was measured from each slide, and the average value was used. The glomerular volume (GV)-ratio was calculated by dividing GV 12 months after transplantation by GV 1-h posttransplantation.

### Statistical analysis

Categorical variables are presented as numbers and percentages, and groups were compared using the chi-square test. Continuous variables were expressed as median, minimum, and maximum values. Groups were compared using Student’s *t*-test. Pearson’s correlation coefficients (*r*) were calculated, and single and multiple regression analyses were performed. Analyses were conducted using SPSS 27.0 (SPSS Inc., Chicago, IL, USA) and SAS software V.9.4 (SAS Institute, Cary, NC, USA). All variables that had a *p* < 0.05 in a single regression model were included in a multiple regression model. A value of *p* < 0.05 was considered statistically significant.

## Results

### Demographic and clinical characteristics

Table 1 shows the demographic and clinical characteristics of the 100 patients. There were no significant differences in background factors, except for total ischemia time, between the developmental cohort and the validation cohort (113 min vs. 85.5 min, *p* < 0.001). The 1-year graft and patient survival rates were 100% in both cohorts.

**TABLE 1:** Demographic and clinical characteristics.

	Developmental cohort (*n* = 79)	Validation cohort (*n* = 21)	*p*-value
**Recipient**			
Age	45 (22–68)	51 (18–72)	0.20
Sex, male	55 (70%)	15 (71%)	0.87
BMI	22.9 (17.6–29.7)	22.1 (16.8–30.4)	0.24
**Background disease**			
Chronic glomerulonephritis	40 (51%)	11 (52%)	0.22
Diabetes mellitus	19 (24%)	2 (10%)	
Hypertension	7 (9%)	1 (5%)	
Others	13 (16%)	7 (33%)	
**Donor**			
Age	60 (32–78)	62 (42–73)	0.79
Sex, male	24 (30%)	10 (48%)	0.11
BMI, kg/m2	23.7 (16.2–28.9)	23.2 (16.6–25.7)	0.56
Body side of the donated kidney, left	69 (87%)	21 (100%)	0.08
Diabetes mellitus, yes	7 (9%)	1 (5%)	0.69
Hypertension	22 (28%)	5 (24%)	0.87
eGFR, ml/min/1.73 m^2^	81.2 (55.3–126.7)	75.8 (57.8–122.0)	0.09
**Transplant**			
ABO incompatible, yes	26 (33%)	8 (38%)	0.66
PEKT, yes	29 (37%)	4 (19%)	0.13
Relationship, parent	43 (54%)	7 (33%)	0.23
Spouse	31 (39%)	12 (57%)	
Sibling	5 (6%)	2 (10%)	
HLA mismatch	3 (0–6)	3 (1–5)	0.79
Warm ischemia time, min	3 (1–16)	4.5 (2–8)	0.06
Total ischemia time, min	113 (67–202)	85.5 (58–120)	<0.001
Bleeding amount, ml	208 (4–2760)	107 (3–871)	0.27

BMI, body mass index; PEKT, preemptive kidney transplant; eGFR, estimated glomerular filtration rate; HLA, human leucocyte antigen.

### Correlation between graft-sim and graft-act and between cortex to recipient weight ratio and simulated graft weight to recipient weight ratio and actual graft weight to recipient weight ratio

**FIGURE 1:**
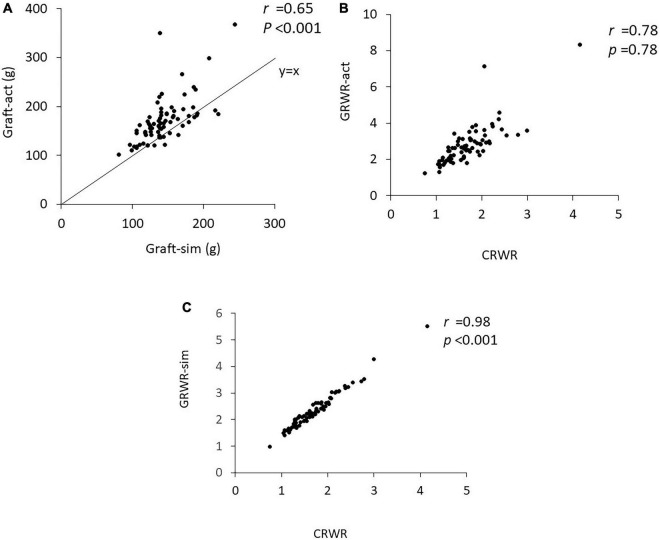
Correlation coefficient between Graft-sim and Graft-act and between CRWR and GRWR-act and GRWR-sim. **(A)** Correlation between Graft-sim and Graft-act. **(B)** Correlation between CRWR and GRWR-act. **(C)** Correlation between CRWR and GRWR-sim.

Graft-sim was positively correlated with Graft-act (*r* = 0.65, *p* < 0.001), and Graft-act tended to be heavier than Graft-sim (Figure 1A). CRWR and GRWR-sim and GRWR-act were positively correlated. CRWR was correlated more strongly with GRWR-sim than with GRWR-act (*r* = 0.98, *p* < 0.001 and *r* = 0.78, *p* < 0.001, respectively, Figures 1B,C).

### Correlation between weight ratio, body mass index ratio, body surface area index ratio, actual graft weight to recipient weight ratio, simulated graft weight to recipient weight ratio, cortex to recipient weight ratio, and urinary protein and postoperative estimated glomerular filtration rate

**FIGURE 2:**
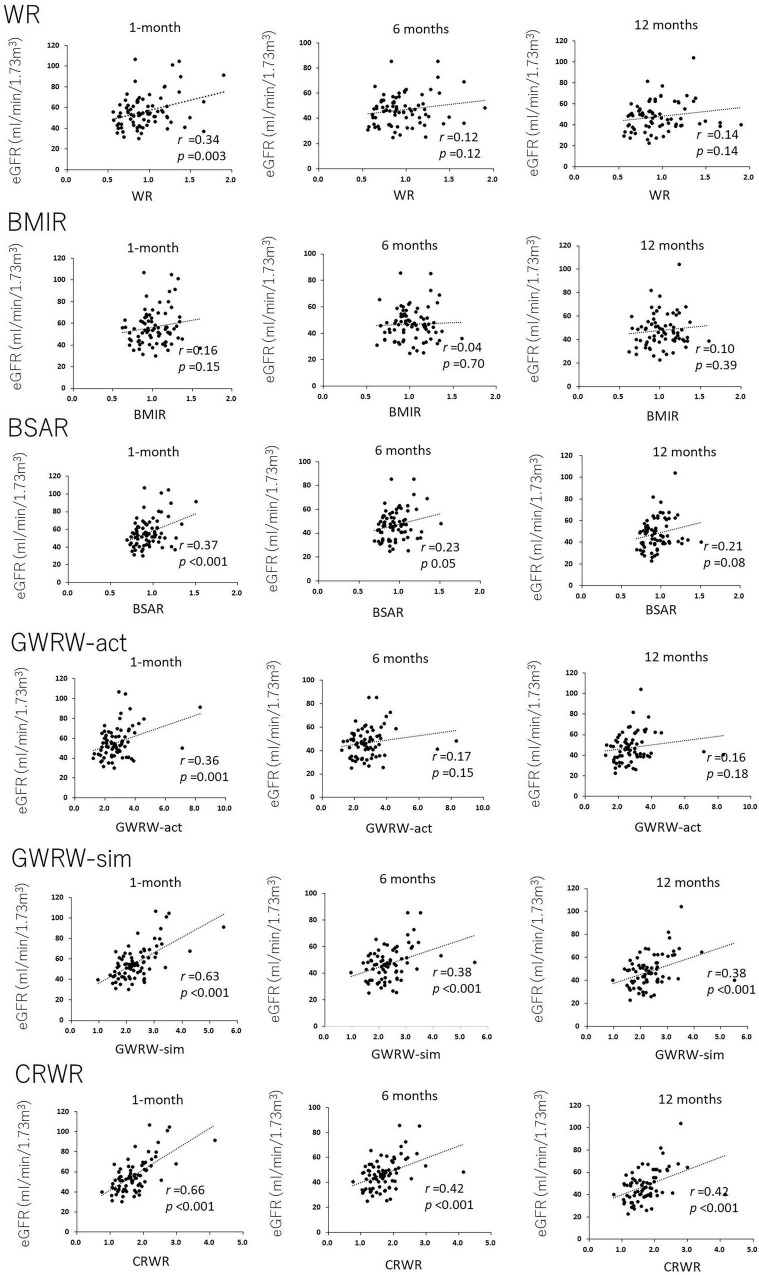
Correlation of donor-recipient size mismatch parameters (WR, BMIR, BSAR, GRWR-act, GRWR-sim, and CRWR) with renal function at 1, 6, and 12 months posttransplantation. The correlation coefficients for CRWR were the highest among the other size mismatch parameters.

Figure 2 demonstrates the correlations between WR, BMIR, BSAR, GRWR-act, GRWR-sim and CRWR and eGFR at 1, 6, and 12 months posttransplantation. WR and BSAR were correlated with postoperative renal function, but the correlation coefficients were low (WR, *r* = 0.34, *p* = 0.003; BSAR, *r* = 0.37, *p* < 0.001 for correlation with eGFR 1-month posttransplantation, respectively). BMIR did not show a significant correlation with postoperative renal function. Correlation coefficients between CRWR and posttransplantation eGFR were generally high compared with those with GRWR-sim and GRWR-act (CRWR *r* = 0.66, *p* < 0.001; GRWR-sim, *r* = 0.63, *p* < 0.001; GRWR-act, *r* = 0.36, *p* = 0.001 for correlation with eGFR 1-month posttransplantation, respectively).

### Correlation between cortex to recipient weight ratio and urinary protein and glomerular volume-ratio

**FIGURE 3:**
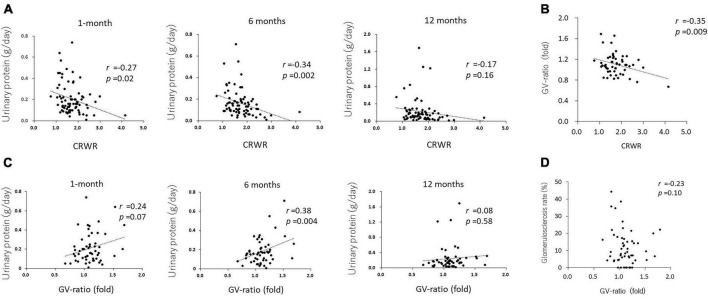
Correlation between CRWR and GV-ratio and urinary protein. **(A)** Correlation between CRWR and urinary protein at 1, 6, and 12 months posttransplantation. **(B)** Correlation between CRWR and GV-ratio. **(C)** Correlation between GV ratio and urinary protein at 1, 6, and 12 months posttransplantation. **(D)** Correlation between GV ratio and the percentage of glomerular sclerosis.

Cortex to recipient weight ratio demonstrated a weak reverse correlation with urinary protein at 1 and 6 months posttransplantation (*r* = –0.27, *p* = 0.02 and *r* = –0.34, *p* = 0.002, respectively, Figure 3A), but not with urinary protein at 12 months posttransplantation (*r* = –0.17, *p* = 0.16).

The GV ratio showed a weak reverse correlation with CRWR (*n* = 56, *r* = –0.35, *p* = 0.009, Figure 3B). The GV ratio demonstrated a positive correlation with urinary protein at 1 and 6 months posttransplantation (*n* = 55, *r* = 0.24, *p* = 0.07 and *r* = 0.38, *p* = 0.004, Figure 3C). On the other hand, the GV ratio showed no correlation with urinary protein posttransplantation or glomerular sclerosis at 12 months posttransplantation (*n* = 55, *r* = –0.08, *p* = 0.58; *n* = 53, *r* = –0.23, *p* = 0.10, Figure 3D).

### Multiple regression analysis using cortex to recipient weight ratio and estimated glomerular filtration rate at 1-month posttransplantation and validation

Single regression analysis showed that donor age (β = –0.33, *SE* = 0.16, *p* = 0.04), donor preoperative eGFR (β = 0.37, *SE* = 0.11, *p* = 0.001), CRWR (β = 20.38, *SE* = 2.67, *p* < 0.001), and preemptive kidney transplant (PEKT, β = 14.34, *SE* = 3.43, *p* < 0.001) were significantly correlated with recipient eGFR at 1-month posttransplantation. In multiple regression analysis, the donor preoperative eGFR (β = 0.23, *SE* = 0.08, *p* = 0.006), CRWR (β = 17.03, *SE* = 2.49, *p* < 0.001), and PEKT (β = 8.96, *SE* = 2.64, *p* = 0.001) became significantly associated with the recipient’s eGFR at 1 month (Table 2). The formula for predicting eGFR at 1 month was expressed as


eGFR(ml/min/1.73m)2

 =0.23×donoreGFR(ml/min/1.73m)2

 +17.03×CRWR+8.96×PEKT

 +5.10(adjustedcoefficientofdetermination=0.54)

**TABLE 2:** Multiple regression analysis related to recipient‘s eGFR (ml/min/1.73 m^2^) at 1 month.

Variable	β	SE	*P*-value
Donor eGFR (ml/min/1.73 m^2^)	0.23	0.08	0.006
CRWR	17.03	2.49	<0.001
Preemptive kidney transplant, yes	8.96	2.64	0.001

eGFR, estimated glomerular filtration rate; CRWR, cortex recipient weight ratio; β, regression coefficient; SE, standard error.

**FIGURE 4:**
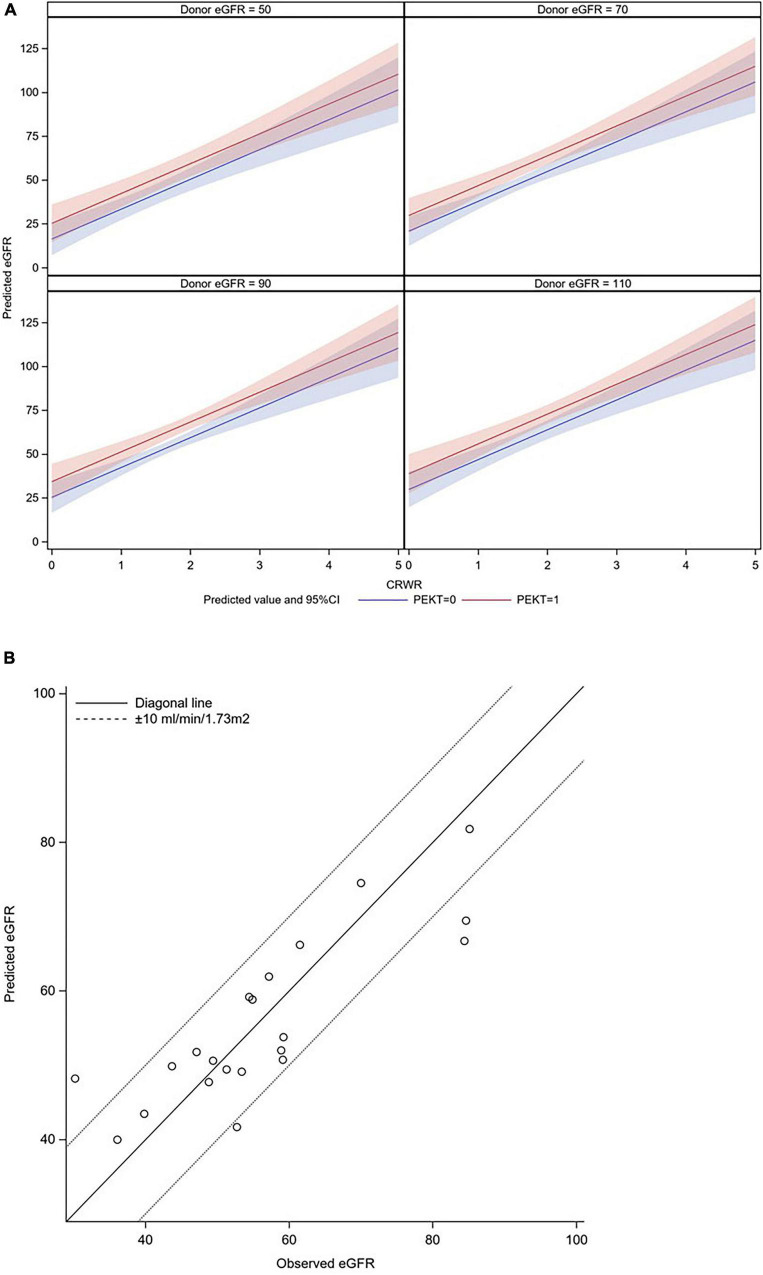
Graphic illustration of the formula for predicting eGFR at 1-month posttransplantation and its validation. **(A)** Graphic illustration of the formula. The donor eGFR is divided into approximately 50, 70, 90, and 110 ml/min/1.73 m^2^. It was possible to calculate the postoperative eGFR for each CRWR according to the presence or absence of PEKT (red line; with PEKT, blue line; without PEKT). **(B)** Validation. In most patients (17/21), the observed eGFR was within a 10 ml/min/1.73 m^2^ margin of the predicted eGFR.

This formula is graphically illustrated in Figure 4A. In this graph, the donor eGFR is divided into approximately 50, 70, 90, and 110 ml/min/1.73 m^2^, and it was possible to calculate the postoperative eGFR for each CRWR according to the presence or absence of PEKT. Furthermore, the formula was validated using the external cohort from the Mito Medical Center (*n* = 21). In more than 80% of patients (17/21), the observed eGFR was within a 10 ml/min/1.73 m^2^ margin of the predicted eGFR, showing that the performance of the formula was good (Figure 4B).

## Discussion

The novelty of this study is that CRWR was a novel size mismatch parameter that directly reflected the “nephron mass” of the allograft and could be calculated form preoperative CT images using CT-volumetric software. CRWR demonstrated the strongest correlation with postoperative early renal function among the representative donor-recipient size mismatch parameters, and it enabled the prediction of early allograft function using a formula.

Factors that influence renal function after LDKT can be divided into immunologic and non-immunologic factors (5). In the 21st century, the introduction of immunosuppressive agents such as calcineurin inhibitors, mycophenolate mofetil, anti-CD25 monoclonal antibody, anti-human thymocyte rabbit immunoglobulin, and rituximab in blood group incompatible transplantation, as well as detailed elucidation of the pathogenesis of various immunologic rejective reactions, have enabled sustained control of immunological factors. Owing to these developments, the short- and mid-term outcomes over the last 20 years have improved dramatically. On the other hand, non-immunological factors, such as nephrotoxicity secondary to immunosuppressants, hypertension, obesity, diabetes, the recurrence of primary disease, donor-recipient size mismatch, and viral infection, affect not only the short-term but also the mid- and long-term outcomes. Controlling these non-immunologic factors to prolong graft survival has received attention from transplant physicians (4). After LDKT, most patients exhibit IGF, in which serum creatinine reaches its nadir within a few weeks posttransplantation. In this period, patients are usually under strong immunosuppression due to the use of anti-CD25 monoclonal antibodies and anti-human thymocyte rabbit immunoglobulin and high serum levels of maintenance calcineurin inhibitors. Thus, renal function in this early period is mostly defined by non-immunological factors, especially the “nephron mass” in the donor graft, reflected by donor age, donor preoperative eGFR, sex mismatch, and donor-recipient size mismatch, that accomplish subsequent baseline graft function (10, 11). In particular, the donor-recipient size mismatch is a direct reflection of the total throughput of the renal graft in the recipient. This can be understood on the basis of two facts: grafts from elderly donors demonstrate declined renal function because of the reduced “nephron mass” in the donor graft secondary to glomerulosclerosis due to aging, and grafts from sex mismatch transplantations, especially female to male transplantations, show worse posttransplantation renal function since female kidneys are smaller in size and have less “nephron mass” in terms of absolute quantity than male kidneys (18, 19).

The correlation between postoperative renal function and parameters such as BMIR, BSAR, WR, and GRWR-act have previously been discussed as indicators of donor-recipient size mismatch (12–16). Several reports have demonstrated some degree of correlation, but none of these indices sufficiently reflect the quantity of the “nephron mass.” Although GRWR-act might be a closer indicator than other parameters, it does not always reflect the “nephron mass” because it includes the weight of perirenal fatty tissue, renal portal vessels, and the ureter. In fact, the results of our study showed that Graft-act (g) was generally heavier than Graft-sim (g), and the strength of the correlation between CRWR and GRWR-act was weaker than that between CRWR and GRWR-sim. Furthermore, GRWR-act was weaklier correlated with early postoperative renal function than GRWR-sim and CRWR. Recently, some studies have reported the usefulness of the calculated renal graft volume, namely, the Graft-sim in our study, in which graft volume was measured by contrast-enhanced MDCT using 3D volumetry. Saxena et al. used MRI-based 3D volumetric software to quantify graft volume and reported that its weight ratio correlated with postoperative eGFR at 6 months and 1 year posttransplantation (16). Yanishi et al. used Synaps Vincent^§^ to quantify graft volumes and reported that GRWR-sim was correlated with eGFR at 1-year postoperation (20). On the other hand, these reports did not compare GRWR-sim with other size mismatch parameters, and it was not clear how reliable GRWR-sim was compared with other parameters. Moreover, these researchers assessed postoperative renal function at the time when renal function can be modified by immunological factors and other non-immunological factors, such as diabetes, obesity, hypertension, and infection. CRWR, as we propose here, directly quantifies the renal cortex where functional glomeruli exist. Because donor non-functional glomeruli, i.e., nephrons with glomerulosclerosis secondary to aging, obesity, infarction, etc., are not contrasted by MDCT, these non-functional nephrons are not counted in CRWR (21). CRWR is more reliable as a surrogate of the “nephron mass” than the GRWR-sim, presented as the total graft volume. This is intuitively understandable from the fact that older donors or obese donors who have a thinning renal cortex on preoperative CT with a sufficient graft weight show worse postoperative renal function than expected. Indeed, in our study, CRWR correlated with early renal function more strongly than GRWR-sim at any time point postoperation.

In this study, eGFR at 1-month posttransplantation was expressed as a linear equation using donor preoperative eGFR, CRWR, and PEKT. This provides preoperative information on the degree to which renal function can be achieved postoperatively with any one donor in the case of multiple potential living donors. Therefore, this formula can be applied as a tool in donor selection. In addition, the ability to predict peak renal function avoids unnecessary fluid replacement or diuretic intervention when postoperative renal function has reached the predicted value, thereby avoiding prolonged hospitalization and wasteful use of medical resources. Conversely, if postoperative renal function is not the predicted value, the involvement of immunologic and other non-immunologic factors should be considered, providing a rationale for invasive interventions such as renal biopsy.

A decreased renal graft survival rate after LDKT has been reported in situations where a small donor kidney is transplanted to a large body size recipient (22–24). This is due to the increased hemodynamic load on a single glomerulus in the renal graft, resulting in a state of hyperfiltration. Chronic hyperfiltration associated with reduced functioning of the nephron mass damages the allograft, initiating a vicious cycle of further reduction in the nephron mass, which causes more significant hyperfiltration, leading to a progressive decline in the GFR, proteinuria, hypertension, and eventually graft failure. This was proposed as the “hyperfiltration theory” by Brenner et al. in who demonstrated the relationship between protein intake and the progression of glomerulosclerosis in small kidneys in animal experiments (25). These compensatory hemodynamic changes could also be a proinflammatory trigger leading to alloantigen-dependent kidney damage (22, 26). The results of our study demonstrated that CRWR was negatively correlated with glomerular enlargement. Namely, as the donor-recipient size mismatch increased, the glomerular size in the allograft increased, implying the pressure overload of each glomerulus. Furthermore, glomerular enlargement demonstrated a positive correlation with proteinuria in the short-term after transplantation, implying glomerular damage. These results support the mechanisms of the “hyperfiltration theory” of donor-recipient size-mismatch transplantation. On the other hand, glomerular enlargement was not directly correlated with urinary protein or glomerular sclerosis at 12 months. The reason for these phenomena might be because in addition to hyperfiltration, glomerular damage and sclerosis at this time could be influenced by other factors, including immunologic and non-immunologic factors such as obesity, diabetes, and viral infection (27).

The limitations of this study are as follows. First, this is a retrospective study of patients at two institutions, and the sample size is small. Furthermore, since the study was conducted only in Asian, the size mismatches may not be as significant as those in other countries. Second, this study did not measure inulin clearance in the early postoperative period; thus, eGFR was used as an outcome measure. The eGFR is generally lower than the true GFR obtained from inulin clearance and therefore may not provide a precise evaluation of postoperative renal function. Third, GV should be evaluated by renal biopsies at each time point, but GV obtained from protocol biopsies at 12 months posttransplantation was used to evaluate postoperative GV altogether. Despite these limitations, given that we for the first time proposed CRWR as a surrogate parameter of the size mismatch that directly reflects the “nephron mass,” this study provides unique implications for the application of this novel indicator in clinical practice.

## Data availability statement

The original contributions presented in the study are included in the article/Supplementary material, further inquiries can be directed to the corresponding author.

## Ethics statement

The studies involving human participants were reviewed and approved by Tsukuba University Hospital and Mito Medical Center Internal Review Boards. Written informed consent for participation was not required for this study in accordance with the national legislation and the institutional requirements.

## Author contributions

KT, KF, and TK collected the data. KT and MG analyzed the data. KT, KF, and MG wrote the manuscript. JU, AH, SH, HN, TO, KYu, and KYa did critical comments on the manuscript. All authors contributed to the article and approved the submitted version.

## Supplementary material

Supplementary Figure 1 Measurement of the renal cortex by 3D CT volumetric software (Synaps Vincent^®^).

Supplementary Figure 2 The glomerular volume (GV)-ratio was calculated by dividing GV 1-year after transplantation by GV 1 h after transplantation.

## Abbreviations

BMI: body mass index
BMIR: body mass index ratio
BSA: body surface area
BSAR: body surface area index ratio
CRWR: cortex to recipient weight ratio
eGFR: estimated glomerular filtration rate
Graft-act: actual graft weight
GRWR-act: actual graft weight to recipient weight ratio
GRWR-sim: simulated graft weight to recipient weight ratio
GV: glomerular volume
IGF: immediate graft function
LDKT: living-donor kidney transplantation
MDCT: multidetector raw CT
WR: weight ratio
3D: 3-dimensional.
