# Supplementary figures and images for: Prediction of early graft function after living donor kidney transplantation by quantifying the “nephron mass” using CT-volumetric software
Source: Front Med (Lausanne). 2022 Oct 28;9:1007175. doi: 10.3389/fmed.2022.1007175 (PMC9649930; doi:10.3389/fmed.2022.1007175)

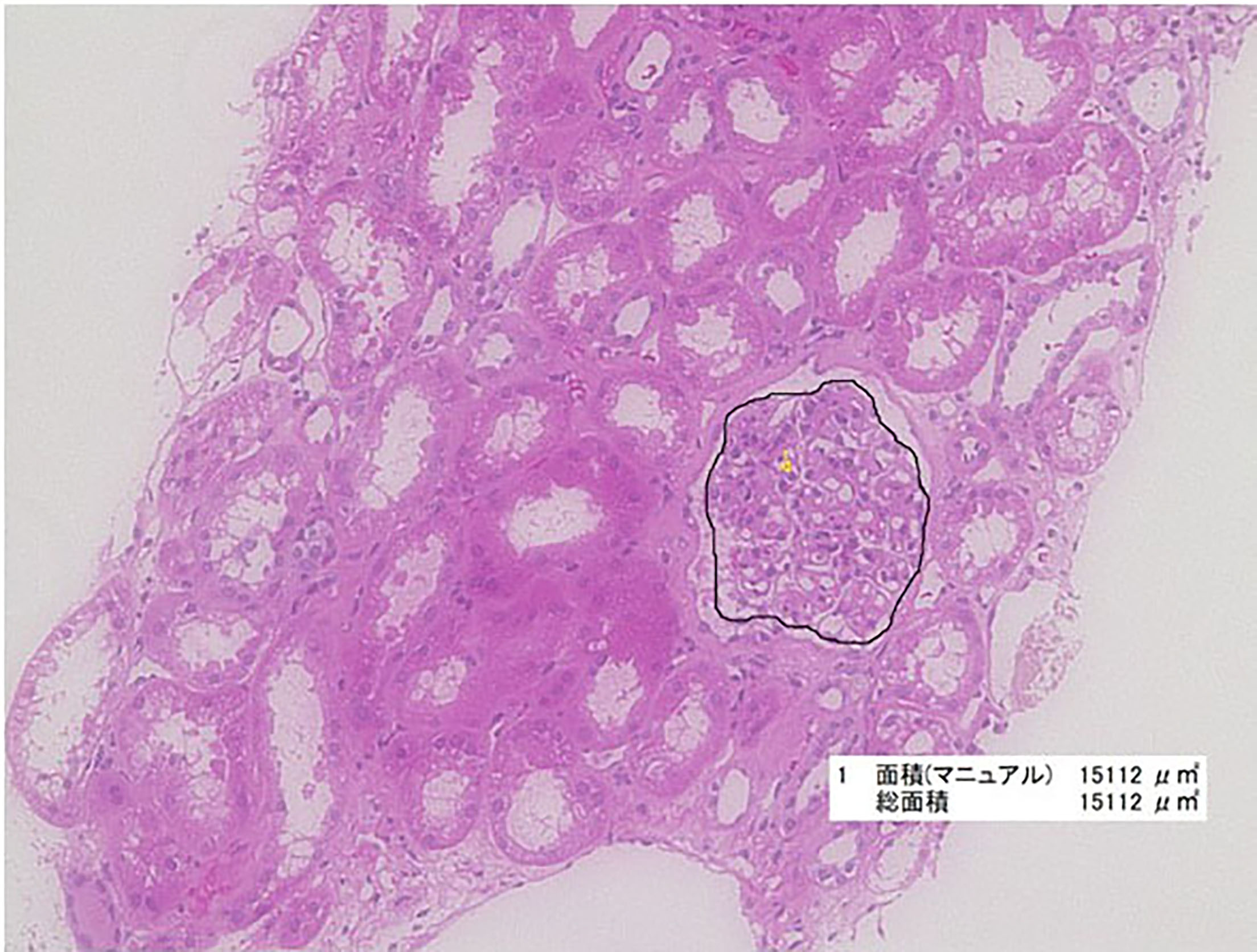

Supplement: Supplementary Figure 1 — Measurement of the renal cortex by 3D CT volumetric software (Synaps Vincent®). [file Image_1.JPEG]

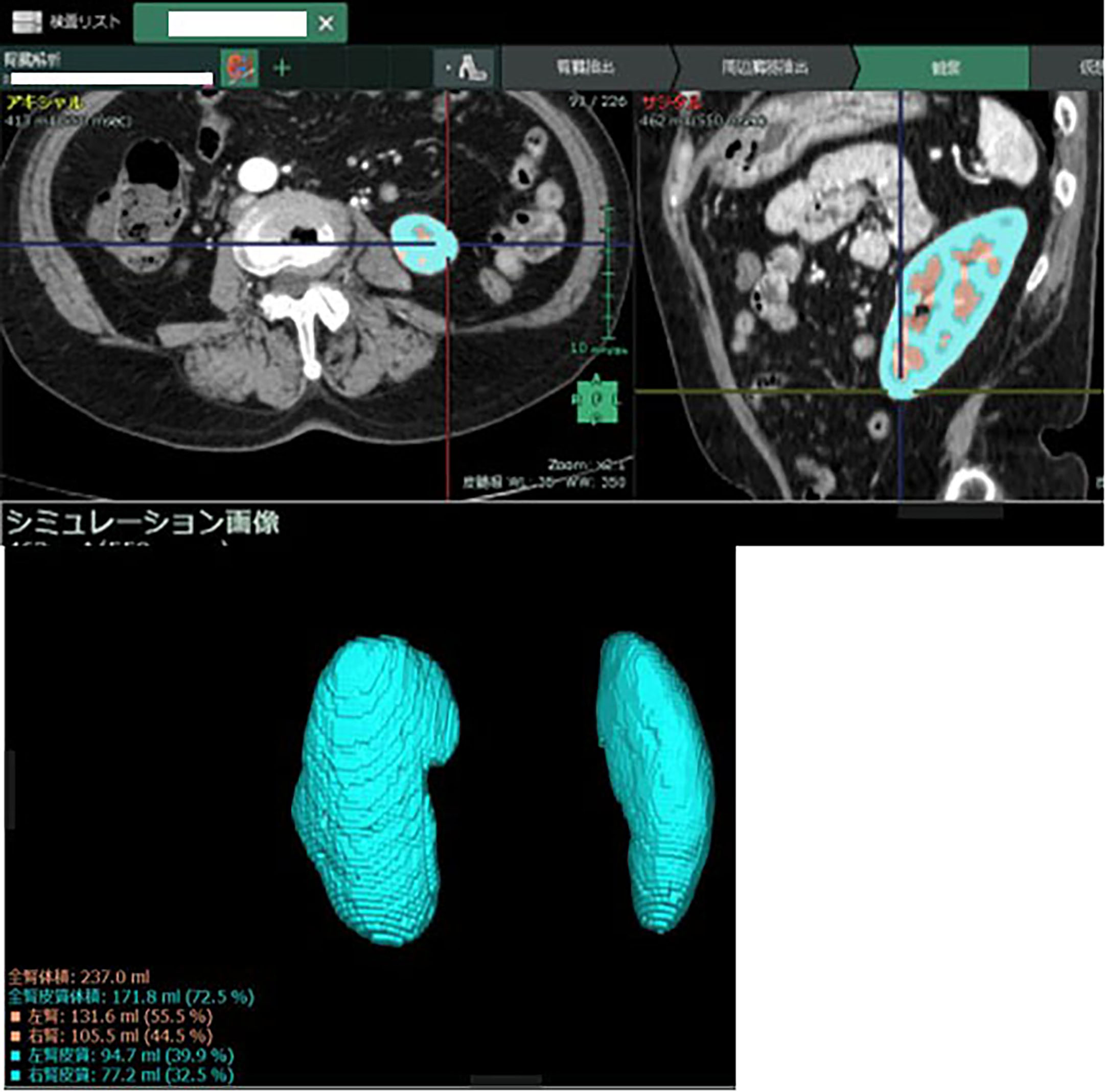

Supplement: Supplementary Figure 2 — The glomerular volume (GV)-ratio was calculated by dividing GV 1-year after transplantation by GV 1 h after transplantation. [file Image_2.JPEG]
